# Development and evaluation of a virtual reality training for emergency treatment of shortness of breath based on frameworks for serious games

**DOI:** 10.3205/zma001598

**Published:** 2023-04-17

**Authors:** Sarah Rickenbacher-Frey, Selina Adam, Aristomenis K. Exadaktylos, Martin Müller, Thomas C. Sauter, Tanja Birrenbach

**Affiliations:** 1 University Hospital Bern, Inselspital, Department of Emergency Medicine, Bern, Switzerland

**Keywords:** COVID-19, emergency medicine, medical education, serious game, shortness of breath, virtual reality

## Abstract

**Background:**

Virtual reality (VR) can offer an innovative approach to providing training in emergency situations, especially in times of COVID-19. There is no risk of infection, and the procedure is scalable and resource-efficient. Nevertheless, the challenges and problems that can arise in the development of VR training are often unclear or underestimated. As an example, we present the evaluation of the feasibility of development of a VR training session for the treatment of dyspnoea. This is based on frameworks for serious games, and provides lessons learned. We evaluate the VR training session with respect to usability, satisfaction, as well as perceived effectiveness and workload of participants.

**Methods:**

The VR training was developed using the established framework (Steps 1-4) for serious games of Verschueren et al. and Nicholson’s RECIPE elements for meaningful gamification. Primary validation (Step 4) was performed at the University of Bern, Switzerland, in a pilot study without control group, with a convenience sample of medical students (n=16) and established measurement tools.

**Results:**

The theoretical frameworks permitted guided development of the VR training session. Validation gave a median System Usability Scale of 80 (IQR 77.5-85); for the User Satisfaction Evaluation Questionnaire, the median score was 27 (IQR 26-28). After the VR training, there was a significant gain in the participants’ confidence in treating a dyspnoeic patient (median pre-training 2 (IQR 2-3) vs. post-training 3 (IQR 3-3), p=0.016).

Lessons learned include the need for involving medical experts, medical educators and technical experts at an equivalent level during the entire development process. Peer-teaching guidance for VR training was feasible.

**Conclusion:**

The proposed frameworks can be valuable tools to guide the development and validation of scientifically founded VR training. The new VR training session is easy and satisfying to use and is effective – and is almost without motion sickness.

## Zusammenfassung

**Hintergrund:**

Virtual Reality (VR) kann einen innovativen Ansatz in der Ausbildung für Notfallsituationen bieten, insbesondere in Zeiten von COVID-19. Hierbei besteht kein Infektionsrisiko, und das Verfahren ist skalierbar und ressourcenschonend. Allerdings sind die Herausforderungen und Probleme, die bei der Entwicklung von VR-Trainings auftreten können, oft noch unklar oder werden unterschätzt. Wir stellen beispielhaft die Durchführbarkeitsbewertung der Entwicklung eines VR-Trainings vor, das für Trainingszwecke zur Behandlung von Dyspnoe vorgesehen ist. Unsere Bewertung basiert auf Rahmenkonzepten für Serious Games und präsentiert die gewonnenen Erkenntnisse. Wir bewerten die VR-Trainingseinheit hinsichtlich der Benutzerfreundlichkeit und -zufriedenheit sowie hinsichtlich der subjektiven Wirksamkeit und Arbeitsbelastung der Teilnehmenden.

**Methodik:**

Das VR-Training wurde anhand des etablierten Rahmenkonzepts (Schritte 1–4) für Serious Games von Verschueren et al. sowie anhand von Nicholsons RECIPE-Elementen für Meaningful Gamification entwickelt. Die primäre Validierung (Schritt 4) erfolgte an der Universität Bern, Schweiz, in einer Pilotstudie ohne Kontrollgruppe mit einer Zufallsstichprobe von Medizinstudierenden (n=16) und etablierten Messinstrumenten.

**Ergebnisse:**

Die theoretischen Rahmenkonzepte ermöglichten die zielgerichtete Entwicklung der VR-Trainingseinheit. Die Validierung ergab für die System Usability Scale (SUS) einen Median von 80 (IQR 77,5-85). Für den User Satisfaction Evaluation Questionnaire (USEQ) lag der Median bei 27 (IQR 26-28). Nach dem VR-Training war ein signifikanter Zuwachs an Selbstvertrauen der Teilnehmenden bei der Behandlung von Patienten mit Dyspnoe zu verzeichnen (Median vor dem Training 2 (IQR 2-3) vs. Median nach dem Training 3 (IQR 3-3), p=0,016).

Zu den gewonnenen Erkenntnissen gehört die Notwendigkeit, medizinische Experten, medizinisches Lehrpersonal und technische Experten gleichermaßen am gesamten Entwicklungsprozess zu beteiligen. Die Anleitung anhand von Peer-Teaching für das VR-Training war praktikabel.

**Schlussfolgerung:**

Die vorgeschlagenen Rahmenkonzepte können wertvolle Hilfsmittel in der gezielten Entwicklung und Validierung von wissenschaftlich fundiertem VR-Training sein. Die neue VR-Trainingseinheit ist einfach und zufriedenstellend in der Anwendung, sie ist effektiv, und darüber hinaus nahezu frei von Anzeichen der Bewegungskrankheit (Kinetosen).

## Background

The COVID-19 pandemic has had a serious impact on the education of medical students in different countries [1]. The disruption of face-to-face training formats has impaired traditional medical education and is likely to have long-term effects beyond COVID-19 [2]. In particular, students criticise the lack of practical teaching [3]. During the current pandemic, it is critically important to provide instruction in dealing with the dominant leading symptom (dyspnoea). It is however very difficult to teach the requisite practical skills and the approach to the patient, especially in acute medicine; and self-protection and the recommended social distancing are crucial. 

Simulation-based training has been shown to be a useful modality to supplement training in real clinical situations, as it allows control over the sequence of tasks offered to learners, provides opportunities to offer support and guidance to learners, prevents unsafe and dangerous situations, and can simulate relatively rare tasks [4]. Traditional simulation-based training sessions are highly resource-intense (personal costs, equipment, locations), may be difficult to adapt to an increasing number of students, and cannot be arbitrarily repeated [5].

To avoid these problems, as well as to fulfil the concept of protection and social distancing during the COVID-19 pandemic, new methods of simulation-based education must be developed and incorporated in the curriculum [6]. These new training formats could involve trainees as peer teachers in the training process, as this would increase trainee involvement while reducing costs for trainers, or might use innovative techniques such as virtual reality (VR). 

Digital transformation in medicine is essential for our digitally experienced students and is also gaining attention within medical schools [7]. VR simulation is a technology that enables the user in real time to explore and manipulate multimedia sensory environments that are both computer-generated and three-dimensional, and thus to gain knowledge that can be applied in clinical practice [8]. There is a growing body of evidence that virtual tools including VR simulation can be engaging and effective for students [8], [9]. Although at the moment VR is mainly used for teaching anatomy or for training technical skills in surgery, it is also being increasingly applied in procedural and differential diagnostic training in acute medicine [8], [10], [11], [12], [13]. In the future, VR might offer benefits for learners and educators, by delivering cost-effective, repeatable, scalable, standardised clinical training, independent of time, location, or instructor, and with intermediate virtual feedback [11]. At the moment, VR training programmes are only slowly being implemented in medical education and have not yet become established despite their theoretical advantages. The reasons for this include the lack of widespread availability of head-mounted displays – both at institutions and in private settings – technical inadequacies of the software programmes and the general lack of digital competencies among students and teachers [5], [6]. 

Moreover, challenges and problems that can arise in the development of VR training are often unclear or underestimated [14]. The problems here can be insufficient preparatory work, different ideas about workflows and technical possibilities and needs, but also general communication problems between doctors, programmers and medical educators. These are familiar in inter-professional teamwork and are linked to differences in training in the different professions. A recent review of serious games in medical education has emphasised the need for theory-based development, as this can help developers to improve the efficiency of their internal processes and provide objective evidence that they are effective [15], [16]. 

We therefore aimed to

1. apply existing theoretical frameworks for the development of a VR training session. And to investigate the lessons learned;
2. evaluate the VR training session with respect to the variables of media use (usability, user satisfaction, immersion, simulator sickness, workload, training efficacy).

## Methods

### Setting

The VR simulation was developed and evaluated from January 2020 until May 2021. The Department of Emergency Medicine, Inselspital, Bern University Hospital, and the Virtual Inselspital Simulation Lab in Emergency Telehealth, Bern University, were responsible for teaching concepts (TB, SR, TCS) and for concepts in emergency medicine (TB, SR, TCS). The technical implementation of the project was provided by a German programming company that is specialised in VR in medical education [https://threedee.de/]. The virtual reality simulation training module aims to treat a patient in a virtual emergency room who is suffering from shortness of breath according to a ABCDE approach [17]. The presented training is part of the “STEP.VR” project (Simulation-Based Training of Medical Emergencies for Physicians and Virtual Reality).

### Framework for development of the VR simulation

The development of the VR simulation was guided by the framework of Verschueren et al. for developing serious games for health [16]. This framework helps in the design of theory-driven, evidence-based serious games for health. Serious games for health are defined as interactive computer applications, with or without significant hardware components, that are challenging, engaging, and which supply the user with expertise that is useful in reality [18].

The framework subsumes five stages (stage 1: scientific foundations, stage 2: design foundations, stage 3: development, stage 4: validation, stage 5: implementation). Each of these has a distinct focus and is implemented by the stakeholders (software developers, medical education specialists, content specialists, and the target audience) within an iterative and repetitive collaborative process. In general, a stakeholder is a person or group that has a vested interest in the course or outcome of a process or project [https://de.wikipedia.org/wiki/Stakeholder].

We describe the development of the simulation (stages 1-3), as well as a pilot evaluation in the target population within a peer-teaching setup (stage 4). We also provide an outlook into the planned implementation (stage 5). 

#### Stage 1: Scientific foundations

##### Target audience and outcome objectives

The target audience and outcome objectives were identified and defined by the medical education and content experts of Bern University Hospital [http://www.profilesmed.ch/]. 

##### Theoretical basis

It was important to have a theoretical basis to guarantee that the development was scientifically sound. The VR training session was therefore developed using an established framework [16] as well as Nicholson's frameworks [19], [20].

##### Content validation

Each step of the process was critically reviewed by the development team, the team of medical education and content experts, together with selected end users and external experts in medical education and clinical treatment. This ensures that the desired objectives are aligned with the relevant instructional design. 

#### Stage 2: Design foundations

##### General design, meaningful gamification and game mechanics 

In accordance with Nicholson, elements for meaningful gamification [20] (RECIPE: reflection, engagement, choice, information, play, and exposition) were applied to help guide the development of the simulation. The RECIPE acronym stands for the following elements:

- **Reflection (R):** This element is intended to connect the training session to emergency events that happen or might happen to the player in real life.
- **Engagement (EN):** The engagement element is related to the creation of a social and engaging learning experience.
- **Choice (C): **Relates to the autonomy the player has within the game. This offers the player the ability to unrestrictedly move in the simulation and to decide on meaningful choices, thus reinforcing his/her autonomy and creating the feeling of being responsible for his/her actions.
- **Information (I): **Serves to provide the key concepts to the player to help him/her to understand the reasons behind the serious game.
- **Play (P): **The “play” element in Nicholson’s approach is defined as “the freedom to explore and fail within boundaries”. The player has freedom in handling the emergency situation and to make choices, which may result, at worst, in a “game over” situation (death of the patient).
- **Exposition (E):** Serves to create a meaningful narrative in the immersive simulation.

In addition to Nicholson’s scheme, other elements of game mechanics (i.e. rewards and feedback) [16] are used in the VR simulation.

##### Design requirements

Design requirements (i.e. language) were tailored to the target audience and the demand for a realistic, immersive emergency environment. We therefore sought regular feedback from the technical experts and potential end users. 

#### Stage 3: Development 

The information gathered in stages 1 and 2 was used to create an effective and engaging educative VR tool in an iterative, repetitive process with the key stakeholders (software developers, medical education specialists, content specialists, and the target audience). The storyboard (see attachment 1 ) was created by the specialists in medical education and content in close collaboration with the technical team, in order to ensure technical feasibility. 

#### Stage 4: Validation

##### Study design and goal

We conducted a prospective feasibility study to investigate the

1. feasibility of peer teaching
2. variables of media use (usability, possible side effects, level of immersion, workload, user satisfaction)
3. training effectiveness

##### Outcome measures

**Variables of media use**

Variables of media use were evaluated according to established questionnaires directly after the training session [21], [22].

Usability was assessed using the System Usability Scale (SUS), which is composed of 10 questions with a five-point Likert attitude scale (range 0 to 100, average score 68) [23], [24] and the After-Scenario Questionnaire (ASQ) [24], which assesses the ease of task completion, satisfaction with completion time and satisfaction with supporting information, on a 7 point Likert scale (total score ranges from 1=full satisfaction to 7=poor satisfaction). 

The User Satisfaction Evaluation Questionnaire (USEQ) has six questions with a five point Likert scale to evaluate user satisfaction (total score ranges from 6=poor satisfaction to 30=excellent satisfaction) [25]. The USEQ and the SUS were used for a differentiated and comprehensive assessment of usability from various dimensions [26]. “Visually-induced motion sickness” was assessed with four items (nausea, headache, blurred vision, dizziness) from the original Simulator Sickness Questionnaire (SSQ) of Kennedy et al. (Likert scale from 1=totally disagree to 5=totally agree) [25]. Presence and immersion in the virtual world were determined according to the 6-item questionnaire developed by Slater, Usoh and Steed (total score ranges from 1=no immersion to 7=full immersion) [27]. 

Perceived subjective workload on a scale from 0 to 100 was assessed using the NASA-Task Load Index [28]. Overstraining is associated with a total score >60, understraining with a total score of <37 [29].

##### Measurement of training effectiveness

To evaluate the perceived training effectiveness, we used the Training Evaluation Inventory (TEI) for the outcome dimension; 17 statements on subjective enjoyment, perceived usefulness, perceived difficulty, subjective knowledge gain and attitudes towards training are assessed on a five point Likert scale ranging from 1=totally disagree to 5=totally agree [30]. Furthermore, we compared the participant’s confidence in dealing with a patient presenting with dyspnoea pre- and post-training (measured on a five point Likert scale (ranging from 1=no confidence to 5=high confidence)).

##### Participants

We included a convenience sample of final year medical students from Bern University (n=16), who responded to a call for participation in our study. All participants attended on a voluntary basis and we provided no remuneration. Written consent was obtained for the study and for publication of the study results.

##### Ethical consent and data storage

The Bern Cantonal Ethics Committee (CEC) considered that this study was exempt from approval (BASEC Nr: Req-2020-00970), as the project is not covered by the Human Research Act, article 2, paragraph 1 in Switzerland.

All methods were carried out in accordance with relevant guidelines and regulations.

Informed Consent to participate was recorded in writing by each participant. The data were collected, analysed and stored in pseudonymised form.

##### Baseline survey

We collected basic sociodemographic data before the intervention (gender, age, need to wear glasses, dominant hand), as well as information on previous experience in the management of patients presenting with dyspnoea, and regular use of computer games and VR simulations.

In addition, participants and peer instructors were able to provide written and oral open feedback on the peer teaching modality.

##### Intervention

The hardware used for this study consisted of an OMEN Gaming Laptop from Bang & Olufsen (HP Development Company, Bremdalvej 8, 7600 Struer, Denmark), as well as the Oculus Rift S tethered head mounted display and controllers (Meta Inc., Menlo Park, California, USA). The VR training sessions were led by a student who was trained as a peer tutor on both the medical content of the simulation and the technical instruction.

All participants first underwent a guided 30-minute training session with a training case with a specific task list – in order to familiarise themselves with the VR environment, with a peer tutor available for instructions as needed. Directly afterwards, the participants underwent the study simulation (case: “shortness of breath/dyspnoea”). 

##### Data analysis 

The statistical analysis was performed in STATA 16.1 (StataCorp, The College Station, Texas, USA). Categorical variables were described through the total number in the categories, accompanied by percentage. As multiple variables were not normally distributed (visually and tested by Shapiro Wilk), the distribution of continuous variables is shown with median and interquartile range (IQR). The Wilcoxon signed rank test was used to to compare the change in confidence pre- and post-training. A p value of 0.05 was considered significant. No adjustment for multiple comparison was performed. 

## Results

### Stage 1 and 2: Scientific and design foundations

#### Target audience

Final year medical students and junior physicians were identified as the primary target population – because of the unmet need for physical simulation programs during medical school, which is exacerbated by social distancing due to the COVID-19 pandemic.

#### Outcome objectives and theoretical basis

The overall outcome was the ability of the target audience to manage an acutely ill patient autonomously and trustworthily for the first half hour, as required in the Swiss catalogue of learning objectives (PROFILES). This included familiarising users with the emergency environment, and the structured clinical ABCDE approach [17] – in the setting of a patient presenting with acute dyspnoea. The learning objectives and their alignment with Nicholsons’ elements of the RECIPE framework [20] are detailed in table 1 (Tab. 1) and attachment 1 . 

##### Reflection (R)

On the one hand, the connection to real life is achieved in VR through the realistic experience of a case that might be observed or experienced in every hospital. On the other hand, virtual debriefing was performed by showing a list of achieved items following the case in the virtual environment and this helps to transfer the learning into practice. There is no personal debriefing after the training with the participant in our specific setting. Depending on the needs of the participants and after embedding in a curriculum, debriefing with a peer tutor or instructor is also possible.

##### Engagement (EN)

The physical condition of the virtual patient reacts to the actual treatment and diagnostic choices in relation to the ABCDE approach (see attachment 1, supplement table 2 ), which helps to generate an algorithmic flow [31], in order to prevent boredom and to constantly stimulate the participant. Creating complex challenges may generate the risk of frustration. To avoid these negative effects, feedback mechanisms and rewards are used extensively (i.e. physical condition improves upon correct actions, which are reflected visually and auditorily. This initiates a diagnostic procedure and promptly generates a medical result, such as taking a blood sample. The results from the blood sample and the correct use of equipment (non-invasive ventilator, ECG, ultrasound) have an engaging effect) [32].

##### Choice (C)

Relates to the autonomy the player has within the game. The correct selection of diagnostic options and thus the application of the correct therapy are decisive for the survival of the patient. As an example, respiratory failure will increase if the patient is not suctioned, does not receive non-invasive ventilation and is not started on inhaled/intravenous therapy. 

##### Information (I)

Our target audience received teaching on theoretical medical principles during the medical curriculum and the practical application of the VR gear and program during peer tutoring session. 

##### Play (P)

In the VR simulation, the student has the opportunity to experience the success and consequences of his medical actions without danger to the patient. The player/learner has the opportunity to restart the simulation until success/rewards (successful treatment of the patient) is achieved. 

##### Exposition (E)

To create a meaningful narrative in the immersive simulation, the experts carefully reviewed the medical content. This scene is part of everyday life in an emergency department and encourages the participant to move, act and treat the patient like an emergency physician but in the immersive world. 

### Stage 3: Development 

All information gathered in stages 1 and 2 was used in the development of the VR simulation, in an iterative, repetitive process in close cooperation with the key stakeholders (software developers, medical education specialists, content specialists, and the target audience). The storyboard was created by specialists in medical education and content, in close exchange with the software developers, in order to help to ensure feasibility. Probably the most fundamental insight during the development phase is the need for constant intensive exchange between the participants of the development team (educators, content experts and technicians) from the very beginning, as different technical languages are spoken and the previous knowledge and mental models of the planned product do not automatically coincide.

For details about the content of the case see attachment 1 ; for the actions necessary for successful treatment, see attachment 1, supplement table 2 ; the evaluation items are described in attachment 1, supplement table 3 . See also a screenshot from the VR application (see attachment, supplement figure 1 ).

### Stage 4: Validation

#### Study sample

In the pilot evaluation, 16 students were included. Baseline characteristics for all included participants (n=16) are detailed in table 2 (Tab. 2).

#### Feasibility of the peer teaching 

Implementation of the VR training sessions as peer teaching sessions was possible and no problems were reported by participants or peer teachers. The peer support during the VR session was highly appreciated by the participants.

#### Variables of media use

The results of the survey of variables of media use are detailed in table 3 (Tab. 3).

#### Training effectiveness

The perceived training effectiveness was measured with the Training Evaluation Inventory and is detailed in table 4 (Tab. 4). We found that the participants’ confidence in treating a dyspnoeic patient increased significantly after the VR training session (median pre-training 2 (IQR 2-3) vs. post-training 3 (IQR 3-3), p=0.016).

## Discussion

We describe (I) the framework-based development of a VR training session for dyspnoea and insights into lessons learned during development. Secondly (II), we present the results from our validation study and these confirms good usability, user satisfaction and VR immersion without relevant side effects. 

### Development (I)

The use of a structured established framework provided important guidance to the development of this VR training. Aspects of gamification and serious games, such as rewards and a playful approach, with the possibility of making mistakes and learning from them, are brought to the attention of the development team through the use of the corresponding frameworks. Results from our structured development – including initial validation – can be used as a starting point or blueprint to develop future VR simulation training sessions within emergency medicine. This focus on medical education and the theoretical basis are in line with the demands of Gentry et al. [15].

During the whole project, intense and continuous collaboration was required between the technical development team, medical content experts and medical educators. This exchange can be challenging, both because of the different languages employed in the specialities involved, but also because of the aspects of game development that are not normally mastered by medical educators or clinicians. In addition, specific priorities (e.g. educational principles from medical educators) or challenges (e.g. problems in technical realisation) in specific fields may often be unclear. We therefore recommend a co-creation approach with clear goals and responsibilities from the very beginning of the planned project and including medical experts, medical educators and technical experts within a cooperation of equals. The use of frameworks can provide guidance and reduce unnecessary detours and undesirable developments and the associated unnecessary use of human and financial resources. However, this effect cannot yet be quantified.

Multiplayer training, although not employed in the present simulation, could further improve the “engagement” component in a VR Simulation. Social engagement – as experienced with multiple players – must be applied with caution, in order to avoid cognitive overload [33]. This cognitive overload has been shown to reduce the effectiveness of training in a similar emergency medicine setting [21]. On the other hand, a team approach is typical and specifically important in emergency medicine [34]. Alternatively, the approach of peer group teaching might contribute to this feeling of engagement and facilitate learning from and with each other. Our VR simulation was tailored to challenge rather than to overwhelm users. The mental workload was measured by the NASA Task Load Index in our validation study and showed that a balanced result was achieved, without excessive or inadequate training. In the future, the aim should be to personalise and adapt the VR simulation to the learner's level of knowledge and performance and thus to avoid frustration. 

### Validation study (II)

The general usability, as measured with the System Usability Scale (SUS), was above average. As satisfaction is the key component of usability [24], we measured user satisfaction with two measurement tools, the After-Scenario Questionnaire (ASQ) and User Satisfaction Evaluation Questionnaire (USEQ) and obtained comparable good results.

Those results confirm the successful development using the framework based approach.

Simulator sickness was minimal with good level of presence and immersion (Slater Usoh Steed). This high degree of immersion could be achieved with our VR training, although the technical possibilities with standard VR hardware are still limited. In the future, therefore, there may be great potential for further technical developments and improvements in immersion. The influence of maximum realism in VR is currently not clear; this is analogous to physical simulation – where there is an ongoing discussion about whether more realistic high fidelity simulation leads to more effective learning than less realistic low fidelity simulation [35], [36]. It is known that the emotional state in VR is influenced by the level of immersion and emotions in VR can translate to emotions in real life [37]. A high experience of presence has been previously highlighted as an indicator of systematic cognitive engagement with the contents of the virtual environment, and is an important predictor of experience-based learning [38].

The overall effectiveness of the training was was good, as measured with the Training Evaluation Inventory and was similar in all tested subgroups (enjoyment, usefulness, difficulty, knowledge gain and attitudes towards training). Confidence in the ability to treat an emergency patient with dysponoea was significantly increased, but is still not sufficient. Our training can therefore be a first step on the way to educate students about emergency situations and help to reduce the perceived lack of preparation for an emergency situation, as well as the fear of treating emergency patients [39], [40]. Nevertheless, integration of this VR training session into an overall multimodal training concept could be useful. We use the VR simulation within a University Emergency Medicine curriculum that includes lecture and physical simulations. In the future, it is also conceivable that a multiplayer VR simulation could be carried out by each student alone from home, in order to enable joint learning in compliance with social distancing and without the disadvantages of travelling and providing space on site.

**Implementation and dissemination (stage 5): **The present publication of the insights gained makes up part of the dissemination process within the development process. Further implementation is currently under evaluation. VR training will be part of a training concept including different modalities of simulation and will most likely include a peer teaching approach according to our presented findings. The final training program is commercially available [https://threedee.de/portfolio/stepvr/]. 

### Limitations

As regards the validation of the VR training, our study has several limitations: The sample size of our validation is limited and no pre-post testing or comparison with other teaching formats was done. Our validation only covers the lower levels of the Kirkpatrick model of training evaluation [41]. Clinical outcomes as higher levels of training effectiveness are notoriously difficult to achieve and will need further research after broad implementation of the training. However, we applied established and validated tools of assessment to all evaluations – of variables of media use, perceived effectiveness and confidence gained. As the choice of methodology for evaluation must be tailored to the intended purpose of evaluation, the TEI, for example, is validated to investigate whether or not students found training sessions useful and whether they gained knowledge, together with other aspects [30]. 

Since most of the participants in our validation study do not use VR regularly, we cannot estimate the influence of the novelty effect [42] on the results presented. This novelty effect may lead to overestimation of the perceived benefits of VR in our study. The novelty effect has been little studied in the field of VR-assisted education in general and needs further research.

Fundamental technical limitations in the currently available VR technology might also influence the success of training. Examples of this are the lack of haptics or the necessary use of controllers.

## Conclusion

The applied frameworks can be valuable tools to guide the development and validation of a VR training session - with good usability, high user satisfaction and high perceived effectiveness but hardly any motion sickness. Lessons learned include the need of involving medical experts, medical educators as well as technical experts on an equal level during the entire development process. 

## Declarations

### Ethics approval and consent to participate

The Cantonal Ethics Committee Berne (KEK) deemed this study to be exempt from approval (BASEC No: Req-2020-00970), as the project is not covered by the Human Research Act, Article 2, Paragraph 1 in Switzerland.

All methods were carried out in accordance with relevant guidelines and regulations.

Informed consent to participate was recorded in writing by each participant.

#### Consent for publication

Written informed consent for publication was obtained from all participants. 

#### Availability of data and materials 

The data were collected, analyzed and stored in pseudonymised form. All data generated or analyzed during this study are included in this published article and its supplementary information files (see attachment 1 ). All methods were carried out in accordance with relevant guidelines and regulations.

#### Authors contributions

SR responsible for conceptualisation, investigation, methodology, writing the original draft and writing, reviewing and editing. SA was responsible for investigation and writing, reviewing and editing. AE was involved in conceptualisation, writing, reviewing and editing. MM was responsible for data curation, formal analysis, writing, reviewing and editing. TCS was responsible for conceptualisation, data curation, investigation, formal analysis, methodology, project administration, supervision, writing the original draft, writing reviewing and editing and contributed equally to this work with TB. TB was responsible for conceptualisation, data curation, investigation, formal analysis, methodology, project administration, supervision, writing the original draft, writing, reviewing and editing and contributed equally to this work with TCS.

#### Funding

The present manuscript is partially funded by the University of Bern.

## Acknowledgements

The authors wish to thank the development team of ThreeDee, Munich, especially Tobias Mühling and Philip Balonier. 

We express our thanks to the Touring Club Switzerland for supporting telehealth at the University of Bern.

## Competing interests

TCS holds an endowed professorship for emergency telehealth at the University of Bern supported by the Touring Club Switzerland. The sponsor has no influence on the research conducted or the decision to publish.

The authors declare that they have no competing interests. 

## Supplementary Material

Online supplement materials

## Figures and Tables

**Table 1:**
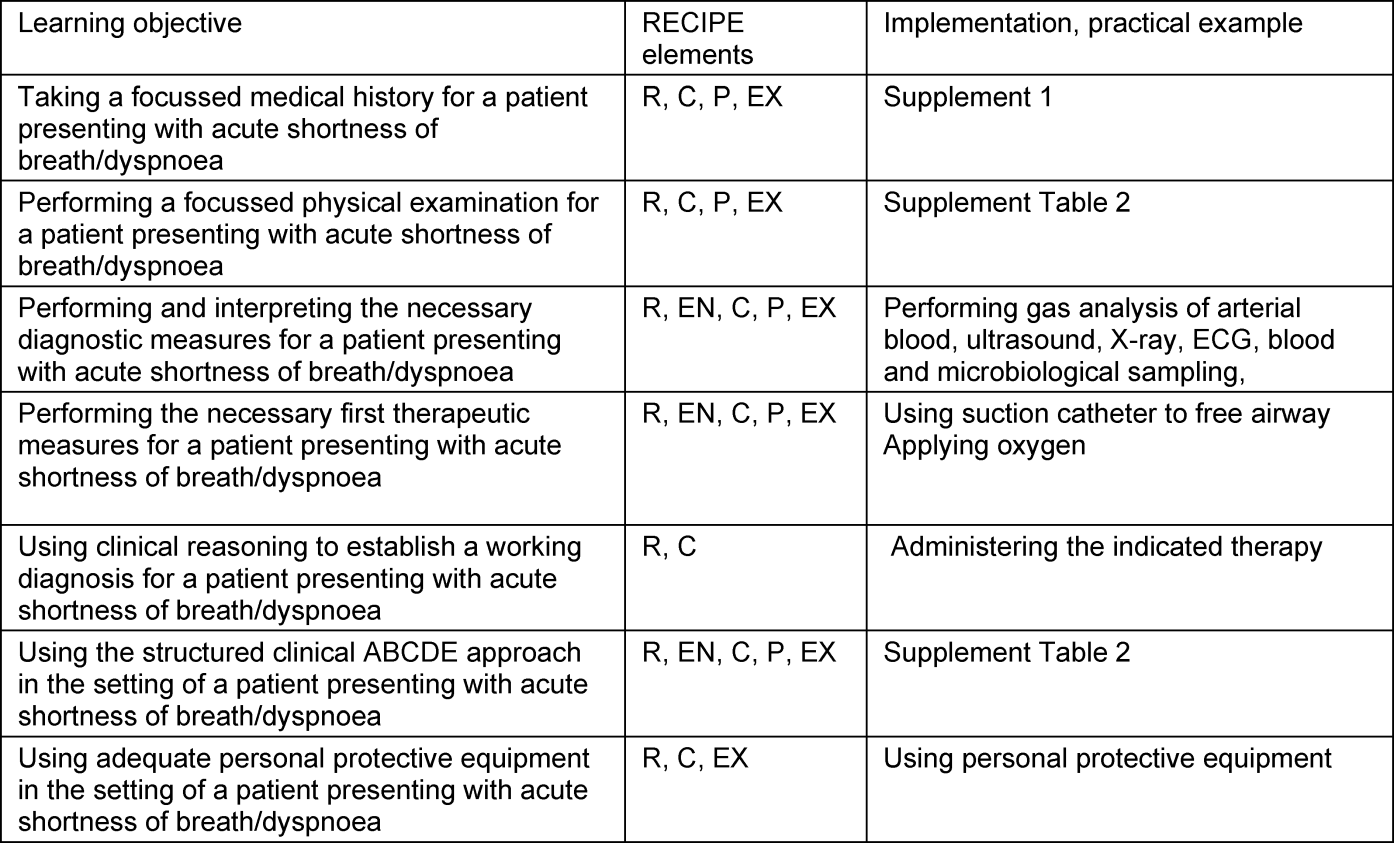
Learning objectives and alignment with the framework RECIPE [21] (R=reflection, EN=engagement, C=choice, I=information, P=play, and EX=exposition), and examples of implementation.

**Table 2:**
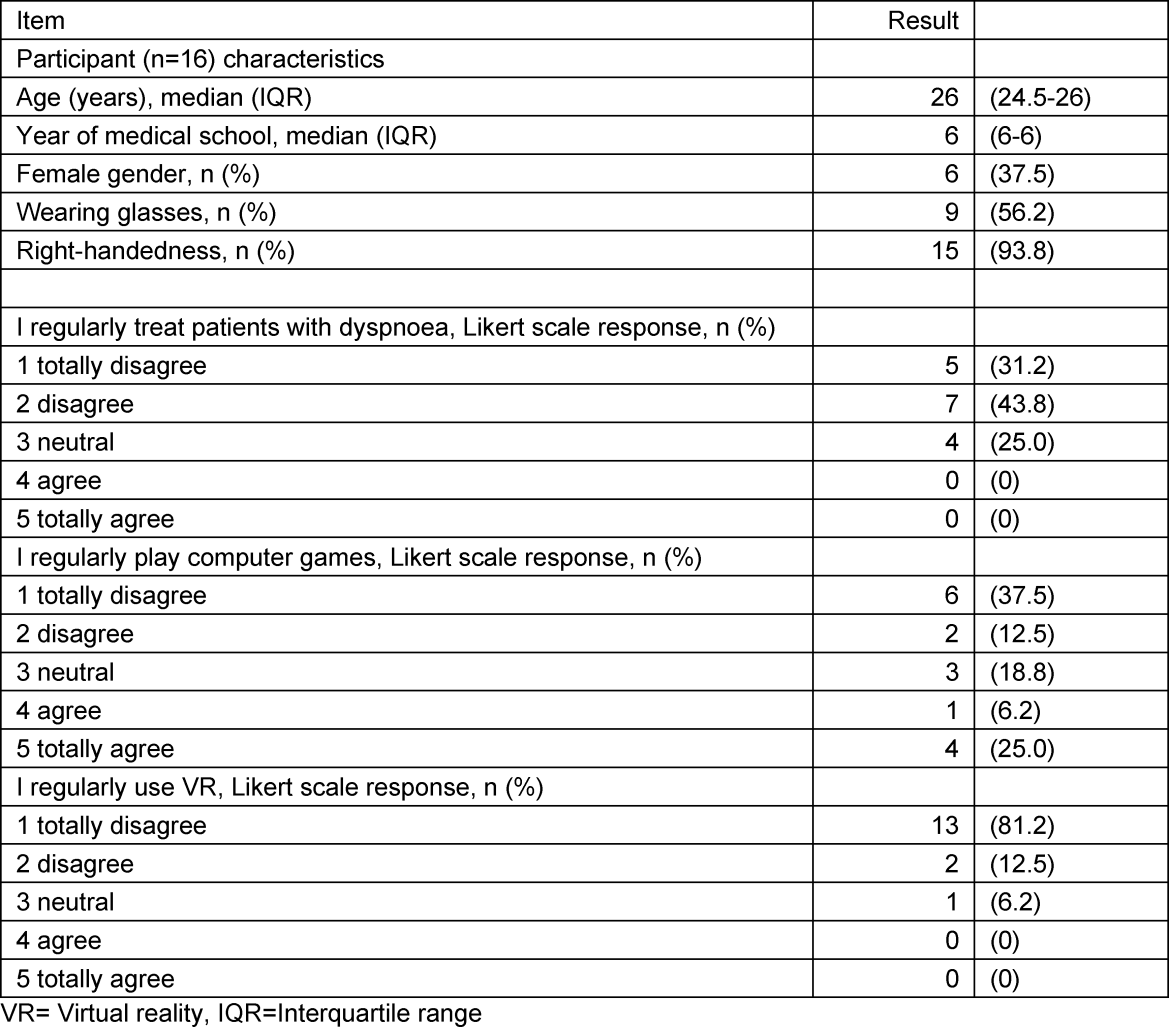
Baseline characteristics

**Table 3:**
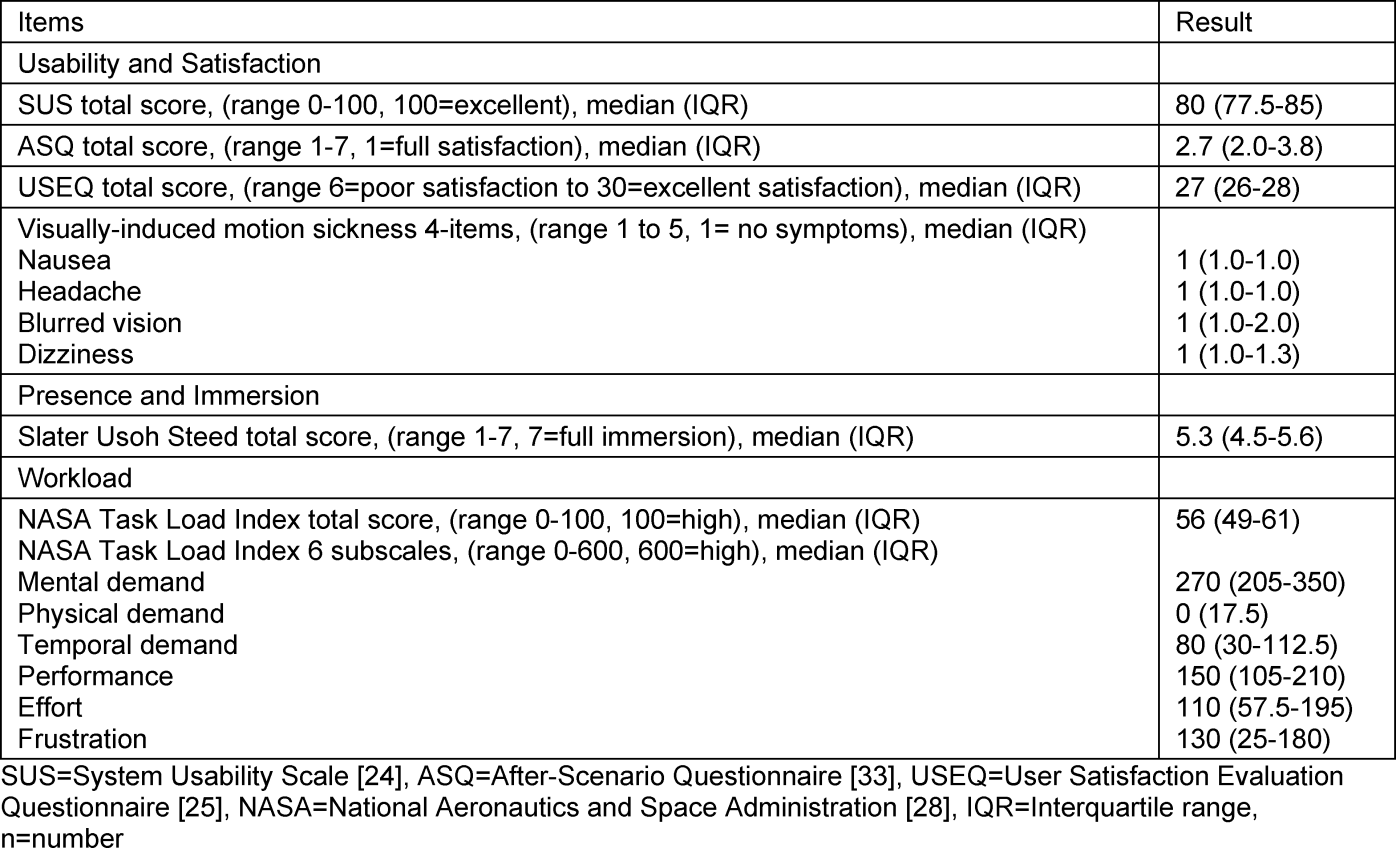
Variables of media use

**Table 4:**
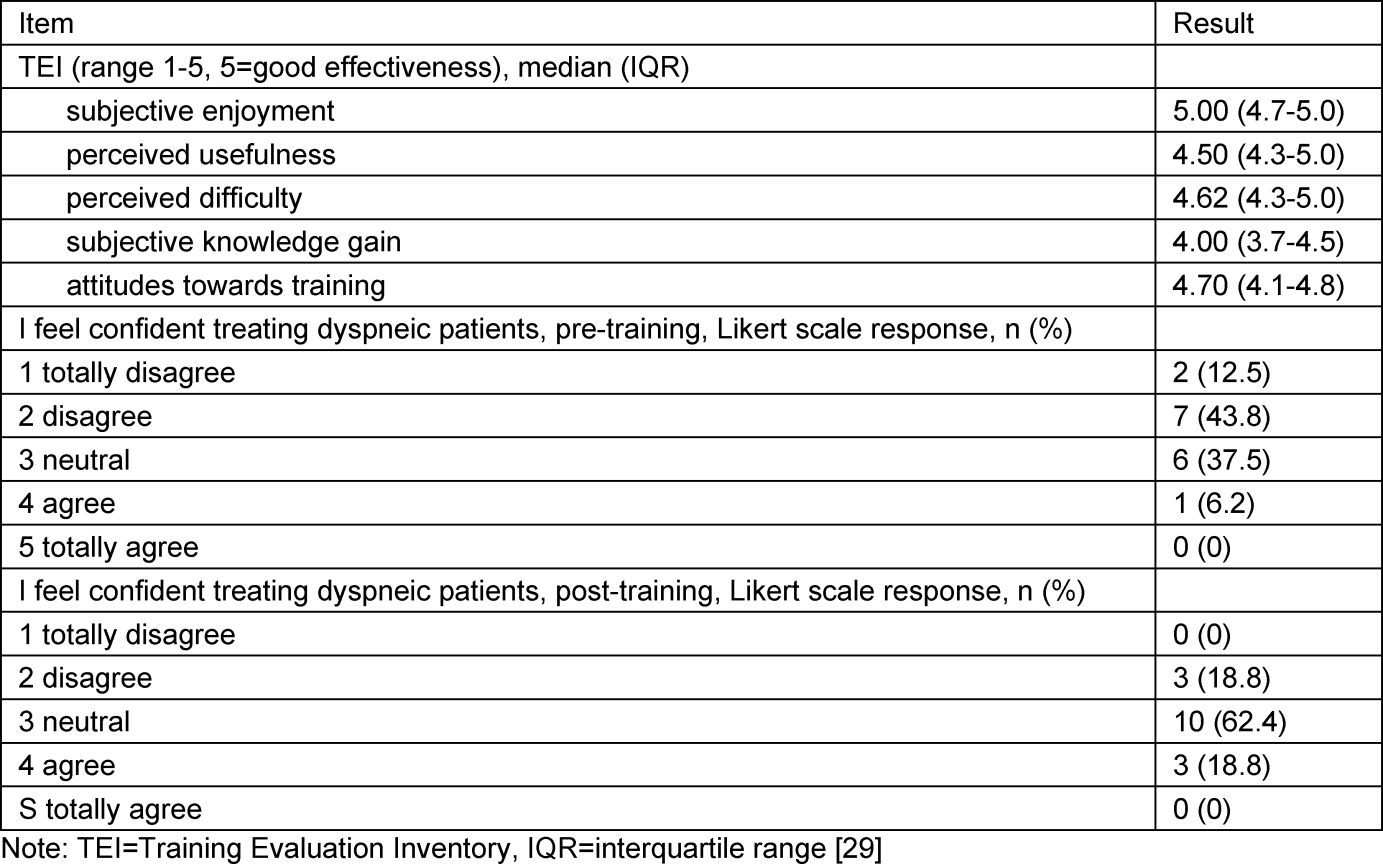
Perceived training effectiveness
