# Supplementary material for: Development and evaluation of a virtual reality training for emergency treatment of shortness of breath based on frameworks for serious games
Source: GMS J Med Educ. 2023 Apr 17;40(2):Doc16. doi: 10.3205/zma001598 (PMC10285370; doi:10.3205/zma001598)
Supplement: Online supplement materials [file JME-40-16-s-001.pdf]

Attachment 1: Online supplement materials

Supplement 1: *Storyboard*

Setting

The scene takes place in an emergency department of a hospital. In front of the patient room, there is protective clothing (disinfectant, coat, gloves, and goggles). The patient lies on a patient bed. All the necessary examination materials for treating the patient are in the drawers. Monitoring and oxygen supply is on the wall. An ECG machine, a non-invasive ventilation machine and an ultrasound machine can be brought into the patient room. A computer for prescribing diagnostic tests and reviewing results of examinations, such as blood results and X-rays, is located at the foot end of the patient bed.

Medical content of the VR training

Case: A 67-year-old male patient presents to the ED with acute shortness of breath. His previous medical history includes an unprovoked deep venous thrombosis three months ago, arterial hypertension, chronic obstructive bronchitis and dyslipidaemia. His medication includes a direct oral anticoagulant, antihypertensives, lipid-lowering agents, and an inhaled bronchodilator. He has a history of smoking (40 pack years) and no allergies.

Initial presentation:

Airway/Cervical-spine: Airway blocked by lots of phlegm

Breathing: Oxygen saturation 82% on room air, breathing rate 40/min, use of accessory breathing muscles

Circulation: Blood pressure 172/90 mmHg, heart rate 122 beats per minute

Disability: Glasgow coma scale 14/15, moves all four extremities, pupils isocor and isoreactive

Exposure/Environment: Body temperature 38.4 °C

Course of the training

The start of the program is set in the doctor's room. The phone is ringing, the nurse is calling for help. The ambulance has dropped off a patient with severe dyspnoea and must leave immediately. The emergency room is very busy. The attending doctor (the participant) is the only doctor available at the moment and the nursing staff has no capacity. The attending physician must treat the patient according to the ABCDE algorithm and initiate the diagnostic

and therapeutically indicated measures. If the actions required for successful treatment are not taken (see supplement table 2) the patient will die after 30 minutes at the latest.

Evaluation

During the treatment part, the dynamic physiology of the virtual patient reflects the success of therapeutic measures comprehensible through changed vital parameters, laboratory values or patient reactions. There is a continuous feedback process. Additionally, there is a final evaluation at the end.

Figure 1

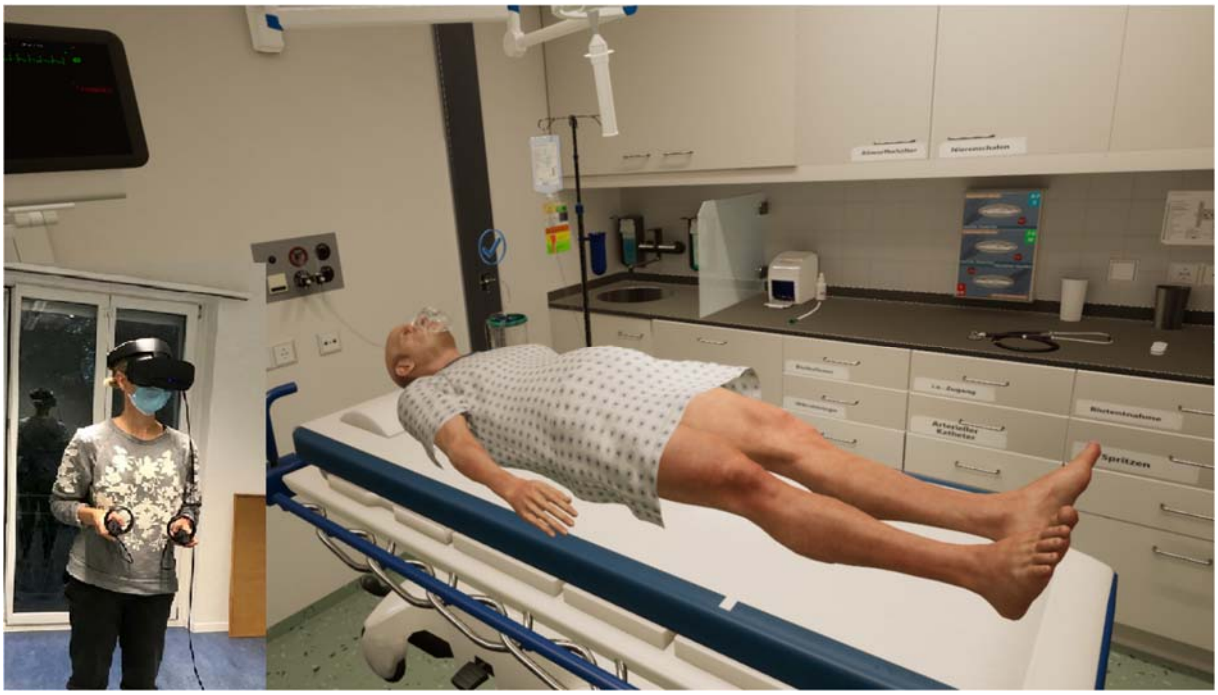

Attachment 1 to Rickenbacher-Frey S, Adam S, Exadaktylos AK, Müller M, Sauter TC, Birrenbach T. *Development and evaluation of a Virtual Reality training for emergency treatment of shortness of breath based on frameworks for serious games.* GMS J Med Educ. 2023;40(2):Doc16. DOI: 10.3205/zma001598

Supplement Table 2: *Actions required for treatment success grouped according to the ABCDE approach*

Airway/Cervical-Spine:<br>- Suction catheter used<br><br>Breathing:<br>- Oxygen administered<br>- Arterial blood gas analysis performed<br>- Point of care ultrasound performed<br>- Chest X-ray performed<br>- Adequate non-invasive ventilation therapy<br>- Adequate medication (morphine/steroids/bronchodilators) administered in correct dosage<br>- No chest/pulmonary angiography performed (pulmonary embolism unlikely)<br><br>Circulation:<br>- Indwelling venous cannula applied<br>- Blood sampling performed<br>- 12-lead electrocardiogram (ECG) performed<br>- Adequate medication (balanced crystalloid solution) administered in correct dosage<br><br>Disability:<br>- Glasgow Coma scale (GCS) scoring performed<br><br>Environment:<br>- Body temperature measured<br>- Adequate microbiological sampling performed<br>- Adequate medication (antibiotics) administered in correct dosage<br>- Adequate use of personal protective equipment

Supplement Table 3: *16 evaluation items*

Interventions
- Suction catheter used
- Arterial blood gas analysis performed
- Complete ultrasound performed
- Chest X-ray performed
- No chest/pulmonary angiography performed (pulmonary embolism unlikely)
- Indwelling venous cannula applied
- Blood sampling performed
- 12-lead ECG performed
- GCS scoring performed
- Body temperature measured
- Microbiological sampling performed

Therapy
- Oxygen administered
- Adequate non-invasive ventilation therapy
- Medication: morphine/steroids/inhalants administered in correct dosage
- Administration of balanced crystalloid solution
- Adequate antibiotic administration

Supplement Illustration 1: *Blueprint of the process stages*

Stage 1: Scientific foundation
- target audience and outcome objectives
- theoretical basis
- content validation

stakeholder ⇩ stakeholder

Stage 2: Design foundations
- general design, meaningful gamification and game mechanics
- design requirements

stakeholder ⇩ stakeholder

Stage 3: Development
- software companys
- storyboard

stakeholder ⇩ stakeholder

Stage 4: Validation
- study design and goal
- outcome measures
- intervention and results

stakeholder ⇩ stakeholder

Stage 5: Implementation and dissemination

Attachment 1 to Rickenbacher-Frey S, Adam S, Exadaktylos AK, Müller M, Sauter TC, Birrenbach T. *Development and evaluation of a Virtual Reality training for emergency treatment of shortness of breath based on frameworks for serious games.* GMS J Med Educ. 2023;40(2):Doc16. DOI: 10.3205/zma001598
